# Polyhexamethylene guanidine phosphate exposure induces abnormal behaviors by disrupting synaptic formation and activity in the cerebral cortex

**DOI:** 10.1186/s12974-026-03832-0

**Published:** 2026-04-23

**Authors:** Moon Yi Ko, Jiwon Choi, Euijun Min, Minjeong Kim, Mi-Kyung Song, Dong Im Kim, Jiyoung Park, Heejin Park, Byoung-Seok Lee, Kyuhong Lee, Sung-Ae Hyun, Minhan Ka

**Affiliations:** 1 Center for Convergence Toxicology Research, Division of Next Generation Non-Clinical Research, Korea Institute of Toxicology, Daejeon, 34114 Republic of Korea; 2 Department of Biochemistry, Chungnam National University, Daejeon, 34134 Republic of Korea; 3 Center for Respiratory Safety Research, Division of Jeonbuk Advanced Bio Research, Korea Institute of Toxicology, Jeonbuk-do, 56212 Republic of Korea; 4 Human and Environmental Toxicology, University of Science and Technology, Daejeon, 34114 Republic of Korea; 5 Center for Biomedical Diagnostic Research, Division of Next Generation Non-Clinical Research, Korea Institute of Toxicology, Daejeon, 34114 Republic of Korea

**Keywords:** PHMG-p, Synapse, Hypoactivity, Anxiety, Spatial learning and memory

## Abstract

**Background:**

Polyhexamethylene guanidine phosphate (PHMG-p), a commonly utilized ingredient in humidifier disinfectants, has emerged as a primary factor contributing to pulmonary damage, asthmatic conditions, and prenatal complications in South Korea. Nevertheless, the extended neurological impacts resulting from PHMG-p exposure have not been adequately investigated. Our objective was to examine the fundamental mechanisms underlying PHMG-p-triggered neurological dysfunction in a murine model after biocide exposure.

**Methods:**

Adult mice aged eight weeks underwent intratracheal instillation (ITI) with either PHMG-p solution (0.9 mg/kg) or saline solution (serving as controls). After a four-week recuperation interval, we examined histopathological features including inflammatory responses and fibrotic alterations in lung tissue. Behavioral performance was assessed through open field testing, Y-maze evaluation, and Rotarod performance tests. Brain tissue molecular characteristics were investigated via RNA sequencing, RT-PCR analysis, and western blot techniques. Cellular composition in neural tissues was determined using immunofluorescence methods.

**Results:**

Four weeks after the exposure, the brains of the mice displayed abnormal neuronal and astrocyte populations owing to activated neuronal death. Notably, exposure to PHMG-p impaired synaptic formation and altered behavioral patterns in the mice. The observed changes appeared to be associated with transfer RNA (tRNA)-mediated neuronal death and synaptic formation, as RNA sequencing-based gene ontology analysis revealed the downregulation of tRNA expression following PHMG-p exposure.

**Conclusion:**

These findings enhance our understanding of the pathophysiological mechanisms triggered by PHMG-p exposure.

**Supplementary Information:**

The online version contains supplementary material available at 10.1186/s12974-026-03832-0.

## Introduction

Polyhexamethylene guanidine phosphate (PHMG-p) serves as an antimicrobial compound that was widely utilized in household humidifier sanitizers throughout South Korea [1]. Data compiled by Korea’s National Apparatus Social Disasters Commission, incorporating both documented and undocumented incidents, indicates that between 1994 and 2011, approximately 20,366 fatalities occurred alongside 950,000 instances of adverse health effects attributed to PHMG-p exposure, with total exposure cases reaching 8,940,000 individuals. Both epidemiological studies and experimental research have shown that exposure to PHMG-p leads to lung inflammation and subsequent fibrotic injury [2, 3]. Additionally, PHMG-p has the potential to trigger acute cardiovascular toxicity, leading to heart failure through severe inflammation, atherogenesis, while pulmonary diseases, such as interstitial lung disease, asthma, and fibrosis, are the most commonly identified outcomes, recent epidemiological surveys among victims have revealed additional symptoms involving the central nervous system (CNS), including memory loss, impaired concentration, depression, and cerebrovascular disorders [4]. This evidence indicates that harmful compounds absorbed through inhalation from domestic consumer products may trigger physiological disruptions extending beyond pulmonary tissues, with possible implications for neurological function. However, to date, the neurological effects of PHMG-p have been reported exclusively through epidemiological observations, and no experimental studies using in vitro or in vivo models have directly investigated the neurotoxic potential of PHMG-p.

Worldwide rates of brain-related dysfunction, developmental cognitive impairments, and progressive neurological conditions have demonstrated substantial escalation in recent decades. Data spanning from 1990 to 2010 revealed dramatic increases across multiple categories: psychological and behavioral disturbances rose by over 37%, Parkinson’s disease incidence climbed 75%, Alzheimer’s disease cases doubled, autism spectrum disorder diagnoses increased 30%, and ADHD identification grew by 16% [5]. The surge in these and additional widespread health crises, especially those affecting neural function, has been associated with contact to toxic environmental substances [6]. Airborne pollutants represent a primary catalyst for inflammatory responses in both pulmonary and cerebral tissues, disrupting standard CNS functioning [7]. These aerial toxicants compromise neurological health through multiple pathways: generating oxidative cellular damage, stimulating immune cell activation in brain tissue, fostering inflammatory processes within neurons, and compromising the selective blood-brain barrier circulation and brain tissue [8].

Synapses are specialized junctional complexes in which axons and dendrites emerge from various neurons [9]. As electrical impulses reach the terminal region of a nerve cell (the transmitting neuron), they stimulate the discharge of chemical messengers that traverse the intercellular gap and attach to binding sites on neighboring nerve cells (the receiving neuron) [10]. Neural connections operate cooperatively across interconnected brain circuits to control nerve cell activity, serving as fundamental components for cognitive functions including acquisition of knowledge, retention of information, and behavioral responses [11, 12]. Impaired synaptic communication may result from internal molecular disruptions within individual cells or modifications in the chemical milieu encompassing these connection sites [7]. Multiple heritable regulatory mechanisms have been identified as crucial controllers of genetic activity that influence adaptive synaptic modifications and information storage processes [13]. Given the lack of experimental evidence on PHMG-p neurotoxicity, the present study aimed to investigate the neurological effects of PHMG-p exposure in a mouse model. Specifically, we examined whether PHMG-p administration induces behavioral abnormalities, cortical neuronal damage, and synaptic alterations, and explored the underlying molecular mechanisms through transcriptomic profiling. Our findings provide the first experimental evidence that PHMG-p disrupts synaptic architecture and gene expression programs in cortical tissue, resulting in behavioral dysfunction.

## Materials and methods

### Animals and housing conditions

Adult female C57BL/6 N mice at eight weeks of age were sourced from Koatech Co., Ltd. (Pyeongtaek, Republic of Korea) and underwent a five-day adaptation period before experimental procedures commenced. Laboratory animals were housed under standardized environmental conditions including temperature control at 23 ± 3 °C, humidity levels maintained between 30 and 70%, and a 12-hour light/dark cycle commencing at 8:00 a.m. All research protocols received authorization from the Korea Institute of Toxicology’s Animal Ethics Review Board.

### Intratracheal exposure of PHMG-p

PHMG-p was procured from SK chemicals (CAS number: 89697-78-9) and prepared using normal saline. For tracheal delivery procedures, female mice underwent random allocation into two study cohorts and received airway instillation as described: the experimental cohort was administered 0.9 mg a.i./kg PHMG-p, whereas animals in the control cohort received equivalent volumes of sterile saline solution employed for compound preparation. The dosage regimen utilized in this investigation was determined from our prior published work [14].

A stock PHMG-P preparation (25% concentration) underwent serial dilution using physiological saline to obtain a final dosage of 0.9 mg a.i./kg, which was subsequently delivered via tracheal instillation of 50 µl per animal utilizing an automated video-guided delivery system (Doobae System, Korea). Before conducting the tracheal procedure, animals received anesthesia through a mobile anesthetic delivery system (L-PAS-02, LMSKOREA, Inc., Seongnam, Korea) featuring an isoflurane delivery mechanism that distributed the anesthetic agent into a sedation enclosure. The laboratory animals underwent exposure to 2.5% isoflurane combined with oxygen (flow rate: 2 L/min) within the chamber, with tracheal compound delivery performed immediately upon achieving adequate anesthetic depth. Behavioral assessments were performed 28 days after PHMG-p administration, followed by euthanasia via CO₂ inhalation and sample collection one week later for subsequent analyses.

### Bronchoalveolar Lavage Fluid (BALF) examination

After sedation procedures, animals underwent left pulmonary ligation followed by systematic irrigation of the right lung compartment through a cannulated airway using three sequential washes with phosphate-buffered saline (PBS, Thermo Fisher Scientific Inc., Waltham, MA, USA). Total cellular enumeration within the recovered bronchoalveolar lavage specimens was quantified using a NucleoCounter device (NC-250; ChemoMetec, Gydevang, Denmark). To facilitate cellular classification analysis, BALF specimens underwent centrifugal preparation via Cytospin methodology (Thermo Fisher Scientific Inc.) and received Diff-Quik staining treatment (Dade Diagnostics, Aguada, Puerto Rico). Cellular identification was accomplished through microscopic examination of distinct cell populations, with 200 total cells assessed per microscopic preparation.

### Tissue morphology examination

Left pulmonary specimens underwent fixation in 10% (v/v) neutral-buffered formalin solution, with subsequent histological preparation performed at the Korean Pathology Technical Center (KP&T, Cheong-ju, Korea). Following standard protocol, paraffin-infiltrated tissue blocks were cut into 4-µm serial sections and underwent staining procedures using Hematoxylin & Eosin (H&E) reagents (Sigma-Aldrich, St. Louis, MO, USA). Processed tissue sections received microscopic examination via light microscopy (Axio Imager M1; Carl Zeiss, Oberkochen, Germany). Individual viewing fields underwent independent assessment to determine the degree of immune cell accumulation, bronchiolo-alveolar cellular proliferation, granulomatous inflammatory responses, and lung tissue scarring. Pathological alterations received evaluation from a qualified histopathologist employing masked assessment protocols as previously established [15, 16]. Histological change severity was categorized using a semi-quantitative grading system from 0 to 4 based on lesion intensity. Grade 0 indicated unaltered normal architecture, whereas grades 1 (minimal), 2 (mild), 3 (moderate), or 4 (severe) represented progressively intensifying degrees or complexity of tissue modifications.

### Behavioral assessments

Behavioral evaluations were conducted during daylight hours by researchers blinded to experimental group assignments. Prior to testing procedures, baseline health parameters including body mass, locomotor patterns, and nutritional intake were assessed. Mice underwent a 4weeks recovery period after exposure of PHMG-p, and then behavioral evaluations were performed for 1 week. The sequence of the behavioral test has conducted in the following orders: Open field assessment, Elevated plus maze assessment (EPM), Y maze assessment, Rotarod assessment. Each behavioral evaluation was conducted on a separate day.

#### Open field assessment

The open field evaluation followed protocols established in our prior work [17]. Individual animals were introduced at the arena periphery within a 71 cm × 86 cm × 56 cm behavioral chamber, with locomotion recorded via video capture for 20-minute sessions. Video analysis was conducted with EthoVision XT 14.0 (Noldus) to determine total distance traveled and mean velocity. Additionally, center zone entries and time allocation within the central region were measured. The testing chamber received 70% ethanol decontamination between mouse.

#### Elevated Plus Maze assessment (EPM)

EPM test was performed using methodology described in our previous study [18]. The EPM is a widely used assay for evaluating anxiety-like behavior in rodents. The parameters measured included the time spent in the open and closed arms. Increased time spent in the closed arms was interpreted as an indicator of heightened anxiety-like behavior. The apparatus featured two unprotected arms (25 cm × 5 cm) and two walled arms (25 cm × 5 cm × 15 cm) arranged in cruciform configuration around a central junction (5 × 5 cm), elevated 50 cm above floor level. Animals were introduced at the center position oriented toward an unprotected arm, with arm exploration monitored for 10-minute periods.

#### Y maze assessment

The Y-maze protocol evaluated novelty spatial preference and spatial cognitive ability for short-term spatial recognition memory following established procedures [19]. The custom acrylic apparatus contained three equivalent arms (35 cm length, 5 cm width, 10 cm height) intersecting at 120° angles from a central hub. Experimental subjects were allocated to different groups to examine spontaneous novelty spatial preference patterns and spatial cognitive. During the training session, mice were allowed to explore two arms while the novel arm was closed. In the trial session, the previously closed novel arm was opened, and spatial novelty preference was evaluated. Sessions were documented using GoPro HERO7 Black Action Camera (GoPro Technology, San Mateo, CA, USA) with subsequent data processing via EthoVision XT 7.0 software (Noldus).

#### Rotarod assessment

Motor coordination was evaluated using rotarod methodology as described [20]. The device featured a textured cylindrical rod (3 cm diameter) positioned 20 cm above ground level (B.S. Technolab, Mouse Rota-Rod Treadmill BR1001), programmed to accelerate from 6 to 60 rpm across a 300-second timeframe. Mice were positioned on the rod during initial constant rotation (6 rpm). Following acclimatization to the rotating surface, formal testing began with automated recording of latency to fall.

### Immunohistochemistry

Brain tissue immunolabeling procedures followed protocols previously described [21]. Primary detection antibodies comprised: mouse anti-NeuN (Abcam, ab104224), rat anti-GFAP (Thermo Fisher Scientific, #13–0300), mouse anti-VGLUT (Synaptic Systems, 135001) and mouse anti-VGAT (Synaptic Systems, 131011). Corresponding secondary antibodies linked to Alexa Fluor fluorescent markers (Thermo Fisher Scientific) were applied for primary antibody visualization.

### Golgi impregnation technique

Dendritic spine visualization employed Golgi-Cox methodology utilizing the FD Rapid GolgiStain Kit (FD Neuro Technologies, Inc., #PK401) based on established procedures [22]. Briefly, whole brains were kept in golgi solution (solution A and solution B mixture) in the dark for 7 days. Then, the brains were transferred to solution C and stored for 3 days. Next, the brains were sectioned coronally (150 μm thick) using a vibratome. And the brain sections were placed in a staining solution (D + E). After washing, the sections were dehydrated stepwise in 50%, 75%, 95%, and 100% ethanol. Finally, the sections were cleared in xylene and mounted using Permount.

### Morphometry

For the morphometric quantification of dendritic spines in mouse brains, images were acquired using Zeiss LSM800 confocal microscopes. Five mice per group were used, and a total of 23 regions of interest across the five mice were analyzed for spine quantification. The total number of regions analyzed per group was described in the figure legends. For synapse quantification, images of 20 different brain sections from more than five mice per condition were obtained by confocal microscopy. The number of synapses was assessed by immunostaining brain sections with antibodies to excitatory (VGLUT) and inhibitory (VGAT) synaptic markers, and by counting VGLUT or VGAT puncta. All images were analyzed using ImageJ (NIH, Bethesda, MD, USA). The calculated values were averaged, and some results were recalculated as relative changes versus control. All counting and classification of dendritic spines were carried out by a single trained individual.

### Western blot analysis

Protein immunoblotting procedures followed established protocols [23]. We prepared brain cortex tissue homogenates using RIPA extraction buffer and quantified protein concentrations with the Pierce BCA Protein Assay Kit (Thermo Fisher Scientific). Protein samples underwent electrophoretic separation on 8%, 10%, or 15% SDS-PAGE polyacrylamide gels before membrane transfer onto PVDF substrates (Thermo Fisher Scientific). Primary antibody panel included: mouse anti-NeuN (Abcam, ab104224), rat anti-GFAP (Thermo Fisher Scientific, #13–0300), rabbit anti-Caspase3 (Cell Signaling, #9662), mouse anti-Bax (Santa Cruz Biotechnology, SC-20067), mouse anti-Bcl2 (Santa Cruz Biotechnology, SC-7382), rabbit anti-SYP (Abcam, ab32594), rabbit anti-PSD95 (Abcam, ab18258), mouse anti-GAD67 (Synaptic Systems, 131011), mouse anti-Gephyrin (Abcam, ab181382) and mouse anti-β-actin (Thermo Fisher Scientific, A5316). Compatible secondary detection antibodies (Thermo Fisher Scientific, 65-6120 and 62-6520) combined with ECL visualization reagent (Thermo Fisher Scientific, 34075) enabled protein detection. Band density quantification was accomplished using ImageJ analysis software from three separate experimental replicates. β-actin expression provided internal loading control for data normalization.

### RNA library construction and genomic sequencing

Sequencing library generation for control and treatment RNA specimens utilized the QuantSeq 3’ mRNA-Seq Library Prep Kit (Lexogen, Inc.) per manufacturer specifications. In summary, 500ng total RNA per specimen underwent processing with oligo-dT primers containing Illumina-compatible sequences at the 5’ terminus, which annealed to RNA prior to cDNA synthesis. After RNA template removal, complementary strand synthesis proceeded using random primers featuring Illumina-compatible adapter sequences at the 5’ end. The resulting duplex libraries received magnetic bead purification to eliminate residual reaction materials. Libraries underwent PCR amplification to incorporate full adapter sequences required for cluster formation. Final libraries received purification to remove amplification components. Massively parallel sequencing was executed as single-end 75 base-pair reads on a NextSeq 500 system (Illumina, Inc.).

### Computational analysis

QuantSeq 3’ mRNA-Seq reads were processed with Bowtie2 [24]. using reference indices built from genome assemblies and transcript databases. Generated alignment outputs facilitated transcript reconstruction, expression quantification, and differential gene identification. Altered gene expression was determined using read counts from singular and multi-mapped alignments through coverage assessment in Bedtools [25]. Read Count (RC) datasets received quantile normalization processing using EdgeR packages within R statistical software (R Development Core Team, 2016) via Bioconductor platform [26]. Gene ontology classification utilized database searches through DAVID (http://david.abcc.ncifcrf.gov/) and Medline repositories (http://www.ncbi.nlm.nih.gov/).

### Statistical evaluation

Data distribution normality was assessed using Kolmogorov-Smirnov testing with inter-group variance comparisons. Statistical evaluation was conducted via two-tailed Student’s t-tests for dual comparisons or one-way/two-way ANOVA with Bonferroni adjustment for multiple group analyses, unless explicitly noted. Data processing utilized GraphPad Prism analytical software (GraphPad Software, Inc.), with outcomes reported as mean (±) standard error of the mean (SEM). Significance thresholds were designated as *p < 0.05, **p < 0.01, ***p < 0.001. Precise P values are detailed within figure captions.

## Results

### PHMG-p administration triggers pulmonary inflammatory responses and tissue scarring in the mice

**Fig. 1:**
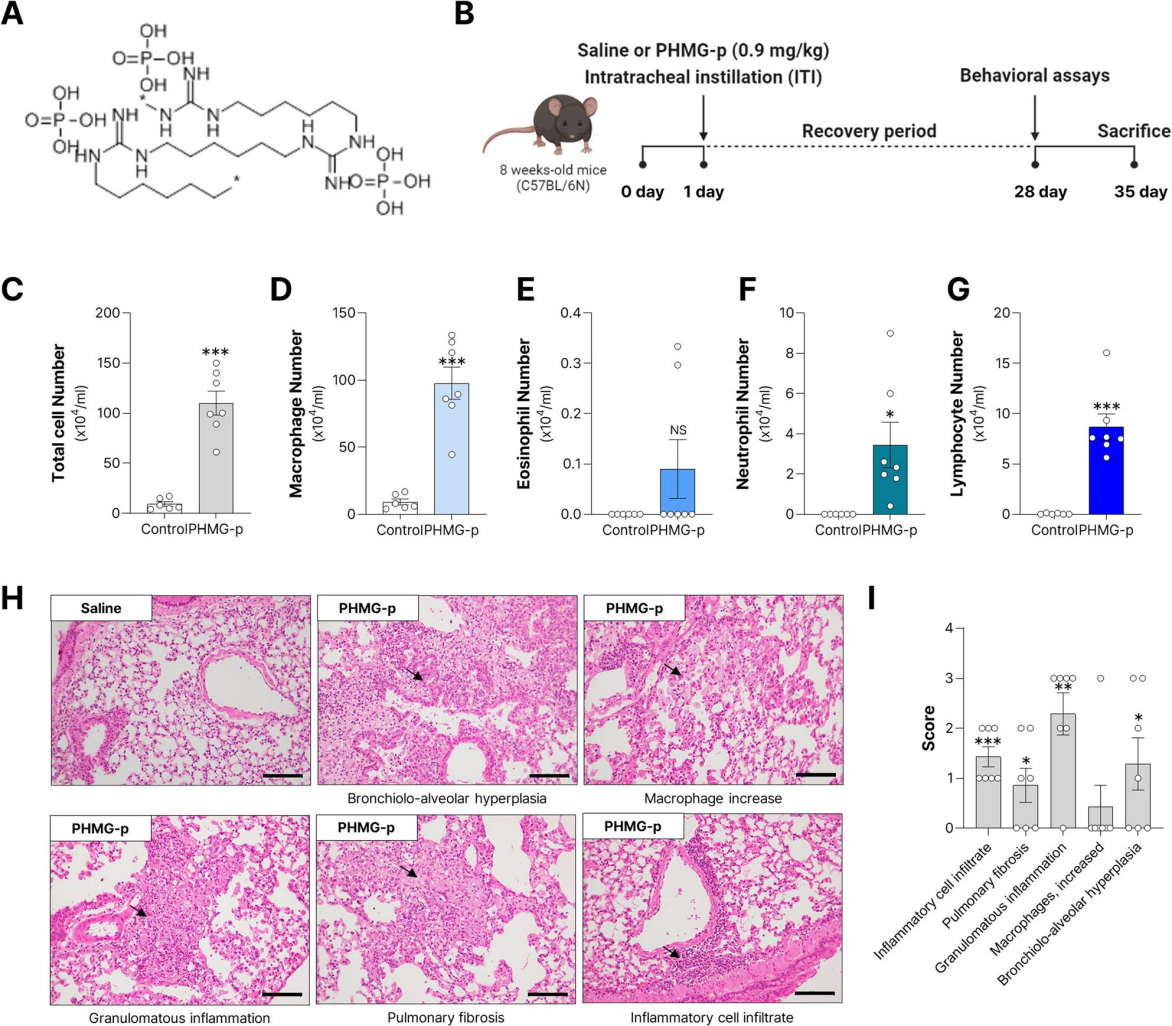
PHMG-p exposure induces lung inflammation and fibrosis in the mice. **A** Chemical structure of PHMG-p. **B** Schematic diagram of the in vivo experimental protocol. **C** Effects of PHMG-p exposure on total cell counts in Bronchoalveolar Lavage Fluid (BALF) of mice (control, *n* = 6; PHMG-p, *n* = 7). **D** Absolute number of macrophages in BALF of control and PHMG-p-exposed mice. **E** Absolute number of eosinophils in BALF of control and PHMG-p-exposed mice. **F** Absolute number of neutrophils in BALF of control and PHMG-p-exposed mice. **G** Absolute number of lymphocytes in BALF of control and PHMG-p-exposed mice (control, *n* = 6; PHMG-p, *n* = 7). **H** Representative H&E-stained lung tissue sections and inflammation scores showing inflammatory cell infiltration, bronchiolo-alveolar hyperplasia, granulomatous inflammation, and pulmonary fibrosis. Scale bars = 500 μm. Arrows indicate bronchiolo-alveolar hyperplasia, granulomatous inflammation, inflammatory cells, macrophages, and fibrotic regions. **I** Quantitative analysis of inflammation scores from PHMG-p-exposed animals shown in panel (**H**), as control mice exhibited no histological alterations (PHMG-p, *n* = 7). Statistical significance is indicated by *p < 0.05 and **p < 0.01, ***p < 0.001

To examine neurological consequences following PHMG-p exposure (Fig. 1A), we first developed a mouse model of respiratory tissue damage. Eight-week-old animals received PHMG-p (0.9 mg/kg) or physiological saline (control) through tracheal delivery (ITI) (Fig. 1B). After completion of the behavioral assessments, we evaluated pathological characteristics of inflammatory processes and fibrotic changes within pulmonary tissues. Saline-treated mice (control) demonstrated minimal cellular alterations in bronchoalveolar lavage specimens (BALF). Conversely, PHMG-p-exposed mice exhibited marked elevation in aggregate cellular counts (Fig. 1C). Analysis revealed significant increases in the absolute numbers of macrophages, neutrophils and lymphocytes in BALF (Fig. 1D, F, G), whereas eosinophil counts did not show a statistically significant increase (Fig. 1E). Tissue morphology assessment was conducted to evaluate pulmonary pathological characteristics after PHMG-p administration (Fig. 1H). Control mouse lung specimens displayed no histological alterations. Examination of H&E-processed tissue sections demonstrated significantly enhanced immune cell accumulation, bronchioloalveolar cellular proliferation, granulomatous inflammatory reactions, and fibrotic tissue formation in PHMG-p-exposed animals relative to control mice. Although macrophage infiltration appeared increased in some PHMG-p–exposed animals, the difference was not statistically significant. (Fig. 1I).

### Behavioral alterations in PHMG-p-exposed mice

**Fig. 2:**
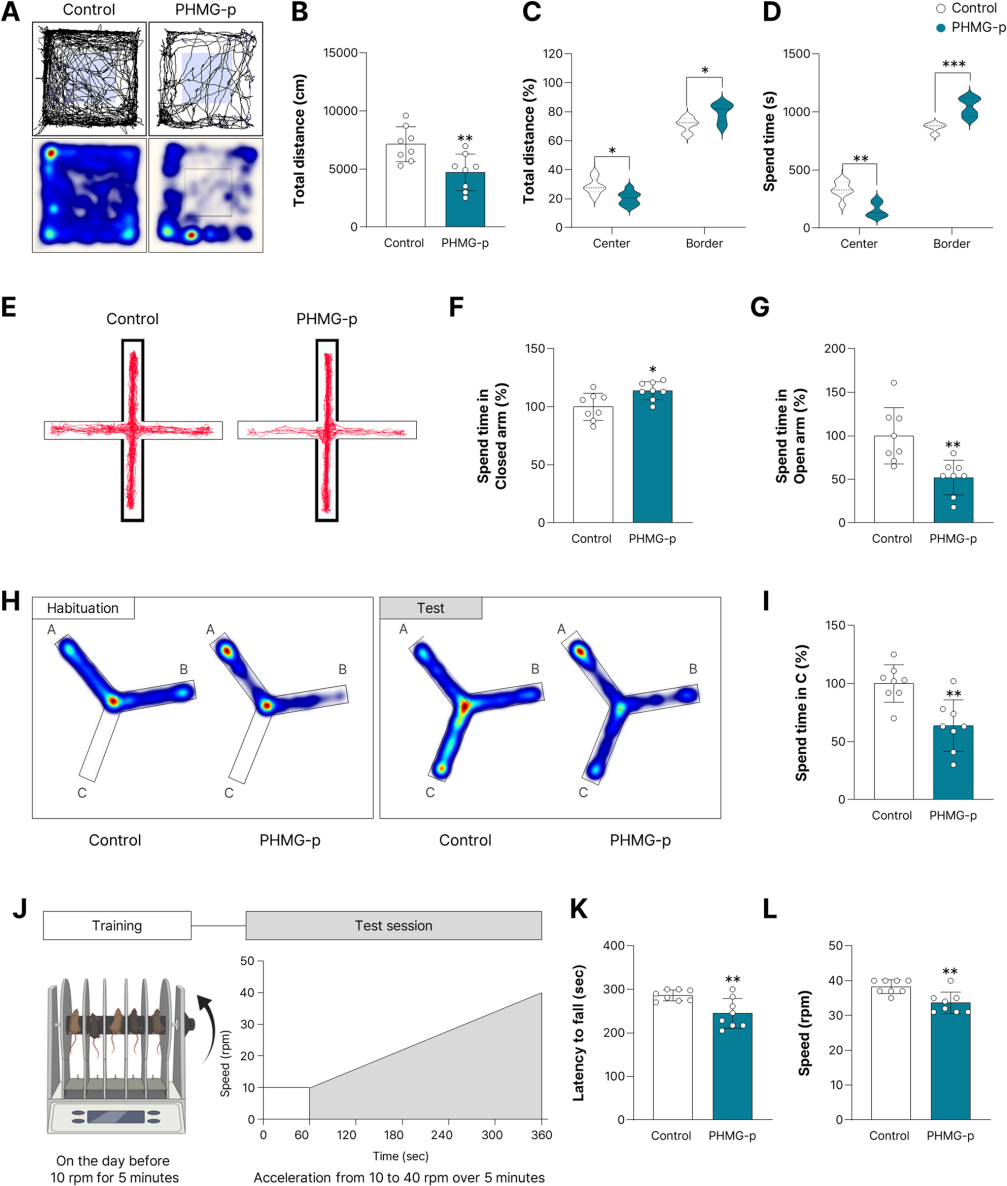
Behavioral alterations in PHMG-p-exposed mice. Eight mice per group were used for all behavioral tests. **A** Representative tracking images from the open field test for control and PHMG-p-exposed mice. Red regions indicate longer time spent in that area. **B** Total distance traveled in the open field test compared between control and PHMG-p-exposed mice. **C** Cumulative distance traveled in the center and border regions of the open field test compared between control and PHMG-p-exposed mice. **D** Cumulative duration spent in the center and border regions of the open field test compared between control and PHMG-p-exposed mice. **E** Representative tracking images from the elevated plus maze test for control and PHMG-p-exposed mice. **F** Time spent in closed arms of the elevated plus maze compared between control and PHMG-p-exposed mice. **G** Time spent in open arms of the elevated plus maze compared between control and PHMG-p-exposed mice. **H** Heatmap data from the Y-maze test for evaluation of short-term memory. Red regions indicate longer time spent in the area. **I** Time spent in the novel arm (C arm) of the Y-maze test compared between control and PHMG-p-exposed mice. **J** Schematic diagram of the rotarod test. **K** Mean latency to fall from the rotarod in control and PHMG-p-exposed mice. **L** Speed at fall from the rotarod in control and PHMG-p-exposed mice. All Statistical significance is indicated by *p < 0.05 and **p < 0.01, ***p < 0.001

To investigate the behavioral responses of mice to PHMG-p exposure, we conducted several behavioral studies using both PHMG-p-exposed mice and control mice after 4 weeks of recovery. Initially, we assessed locomotor patterns within an unfamiliar setting through open-field assessment. PHMG-p-exposed mice demonstrated a significant 34% reduction in overall ambulatory distance relative to control mice (Fig. 2A and B). Additionally, to evaluate anxious behaviors in the animals, we quantified locomotion within and duration of occupancy in the central arena region. These parameters showed respective decreases of 29% and 53% in PHMG-p-exposed mice compared to controls (Fig. 2A, C and D). We validated anxious behavioral responses utilizing elevated plus maze assessment. PHMG-p-exposed animals demonstrated a 14% increase in duration within enclosed maze sections relative to controls (Fig. 2E and F). In contrast, these mice exhibited 48% reduced occupancy time in unprotected arms compared to control mice (Fig. 2E and G). These findings indicate that PHMG-p administration selectively triggers anxiety-related behavioral responses in mice.

Next, we assessed novelty spatial preference and spatial cognitive ability in animals through Y-maze testing. PHMG-p-exposed mice demonstrated 36% reduced exploration time within the unfamiliar arm relative to controls (Fig. 2H and I). These observations suggest that PHMG-p administration disrupts cognitive memory functions in mice. Finally, we examined baseline motor coordination and locomotor capabilities in PHMG-p-exposed animals versus controls through rotarod assessment (Fig. 2J). Time to rod displacement was markedly reduced in PHMG-p-exposed mice compared to control groups (Fig. 2K). Additionally, the rotational velocity at which PHMG-p-exposed animals lost grip was considerably lower than controls (Fig. 2L). Overall, these results suggest that PHMG-p administration selectively promotes anxiety-related behavioral patterns while compromising cognitive memory processes and motor performance in mice.

### Altered neuronal and astrocyte populations in PHMG-p-exposed mice

**Fig. 3:**
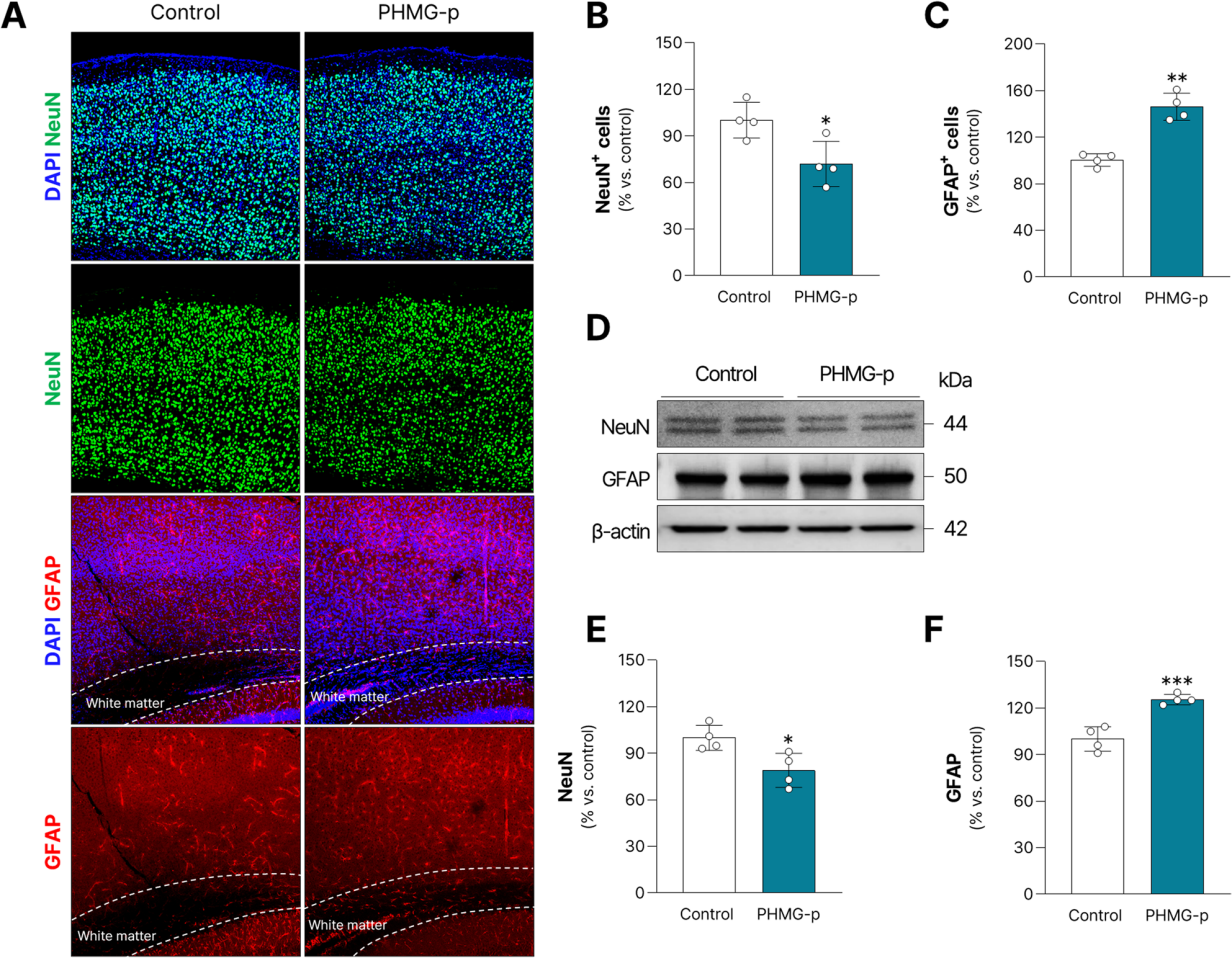
Altered neuronal and astrocyte populations in PHMG-p-exposed mice. **A** Representative images showing NeuN and GFAP expression levels in control and PHMG-p-exposed mice. **B**, **C** Quantitative analysis of data from panel (**A**) (*n* = 4 per group). **D** Representative Western blot images demonstrating NeuN and GFAP protein levels in control and PHMG-p-exposed mice. **E**, **F** Quantitative analysis of data from panel (**D**), normalized to β-actin (*n* = 4 per group). All Statistical significance is indicated by *p < 0.05 and **p < 0.01, ***p < 0.001

We established that PHMG-p administration resulted in aberrant behavioral responses in mice. To precisely determine the underlying causes of these behavioral changes, we examined PHMG-p effects on cortical neuronal populations and glial cell numbers within cerebral tissue. We conducted immunohistochemical analysis using NeuN, a biomarker for identifying mature neurons. Our findings revealed a 20% reduction in neuronal counts within PHMG-p exposed mice relative to controls (Fig. 3A and B). Next, we performed immunohistochemical staining for GFAP, an indicator of astrocytic activation. Results showed a 46% increase in GFAP-immunoreactive cells in PHMG-p-exposed mice compared to control groups (Fig. 3A and C). Furthermore, we analyzed cortical protein extracts via immunoblotting using NeuN and GFAP-specific antibodies. Consistent with histological findings, we documented a significant 21% reduction in NeuN expression levels in PHMG-p-exposed mice versus controls (Fig. 3D and E). Conversely, GFAP expression was elevated by 26% in PHMG-p-exposed mice compared to controls (Fig. 3D and F). These data indicate that PHMG-p exposure promotes neuronal depletion and astrocytic reactivity in mice.

### Aberrant synaptic architecture in PHMG-p-exposed mice

**Fig. 4:**
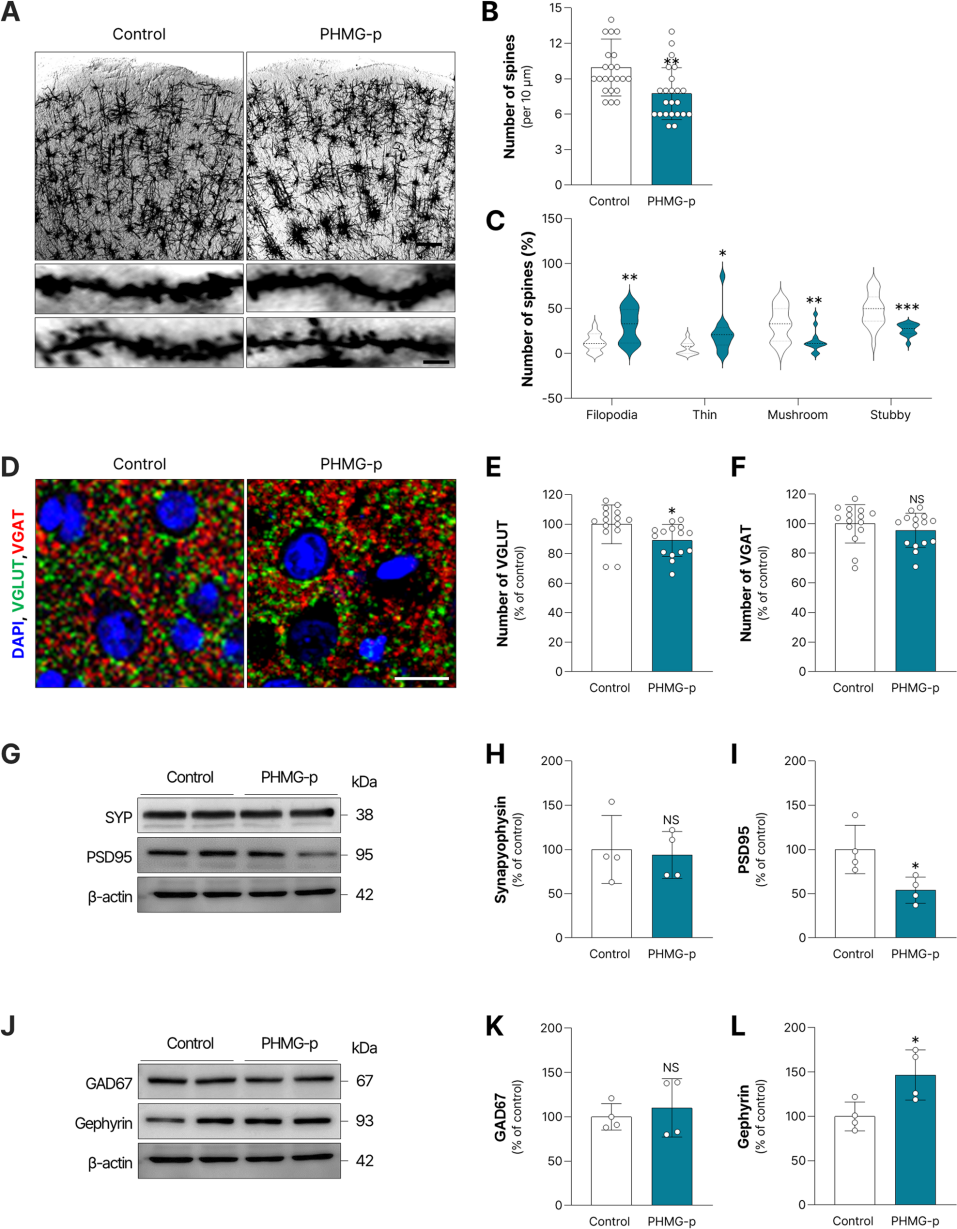
Synaptic alterations in PHMG-p-exposed mice. **A** Representative Golgi-stained images of control and PHMG-p-exposed mice. Scale bars: 50 μm. **B** Quantitative analysis of data from panel (**A**) showing dendritic spine density (*n* = 5 per group; a total of 23 regions of interest across five mice were analyzed). **C** Representative images of dendritic spine morphology and quantitative analysis of data from panel (**A**) showing the distribution of each spine type. **D** Representative images showing VGLUT1 and VGAT expression levels in control and PHMG-p-exposed mice. Scale bars: 10 μm. **E**, **F** Quantitative analysis of data from panel (**D**) (*n* = 5 per group; a total of 15 regions of interest across five mice were analyzed). **G** Representative Western blot images demonstrating SYP and PSD95 protein levels in control and PHMG-p-exposed mice. **H**, **I** Quantitative analysis of data from panel (**G**), normalized to β-actin. **J** Representative Western blot images demonstrating GAD67 and gephyrin protein levels in control and PHMG-p-exposed mice. **K**, **L** Quantitative analysis of data from panel (**J**), normalized to β-actin (*n* = 4 per group). All Statistical significance is indicated by *p < 0.05, **p < 0.01, and ***p < 0.001. NS: not significant

To examine PHMG-p effects on dendritic protrusion development within cerebral cortical regions, both PHMG-p-administered and control animals underwent dendritic spine evaluation via Golgi impregnation methodology. PHMG-p-exposed animal brains exhibited 22.2% fewer dendritic protrusions in cortical pyramidal neurons compared to controls (Fig. 4A and B). Dendritic protrusions are morphologically categorized as filopodia, thin, mushroom, or stubby variants [27] according to structural characteristics. We subsequently characterized dendritic spine subtypes within cortical pyramidal neurons from both control and PHMG-p-exposed mice. PHMG-p-exposed cortical pyramidal neurons demonstrated substantial reductions in mushroom and stubby spine populations relative to control neurons (Fig. 4C). Mushroom and stubby spine densities in PHMG-p-exposed cortical pyramidal neurons were reduced by 56% and 45%, respectively, compared to control neurons (Fig. 4A and C). However, filopodial spine numbers increased by 254% in PHMG-p-exposed cortical pyramidal neurons (Fig. 4A and C). These observations indicate that PHMG-p administration causes dendritic spine architectural abnormalities in cortical pyramidal neurons within cerebral cortical tissue.

To examine PHMG-p influences on cortical synaptic development, we conducted immunofluorescence labeling using VGLUT1 antibodies for excitatory synapse identification and VGAT antibodies for inhibitory synapse detection. VGLUT1-positive puncta showed an 11% reduction in PHMG-p-exposed cortices versus control specimens (Fig. 4D and E). Notably, PHMG-p administration did not modify cortical VGAT puncta density (Fig. 4D and F). Protein immunoblotting utilized antibodies against synaptophysin and PSD95 for pre-excitatory and post-excitatory synaptic labeling, respectively. Synaptophysin expression remained unchanged between PHMG-p-exposed and control cortical samples (Fig. 4G and H). However, PSD95 levels decreased by 45% in treated animals (Fig. 4G and I). Finally, we analyzed GAD67 and gephyrin proteins, which mark pre-inhibitory and post-inhibitory synapses, respectively. GAD67 expression showed no significant differences between PHMG-p-exposed and control cortical tissues (Fig. 4J and K). Conversely, gephyrin levels increased by 47% in PHMG-p-exposed mice compared to controls (Fig. 4J and L). These data collectively establish that PHMG-p treatment leads to structural damage in both dendritic spines and synaptic connections throughout the cerebral cortex.

### Transcriptomic alterations in PHMG-p-exposed mice

**Fig. 5:**
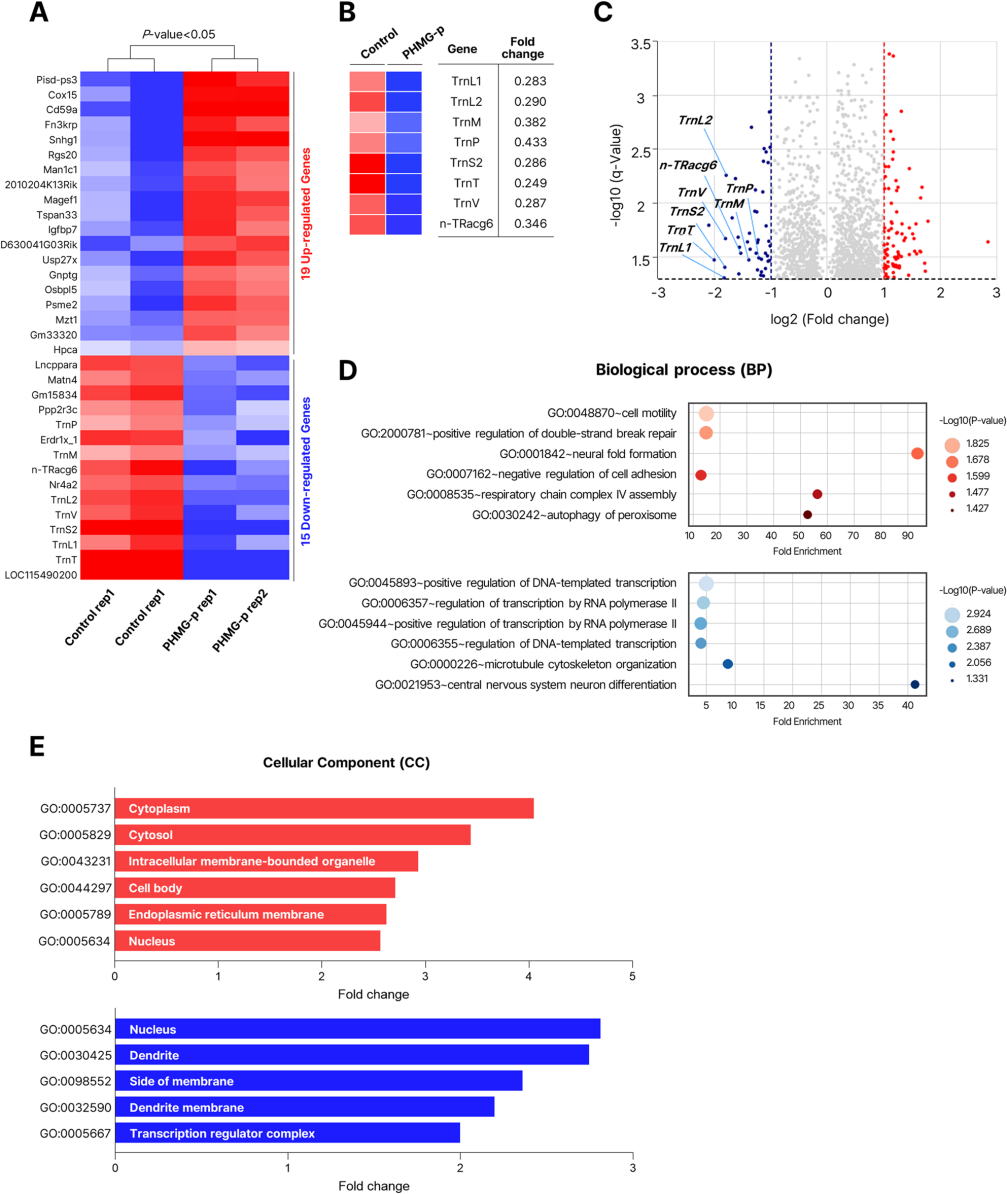
Changes in gene expression profile of PHMG-p-exposed mice. **A** Heatmap comparing differences in gene expression in brain cortices between PHMG-p-exposed mice and control mice. **B** Heatmap of differentially expressed genes associated with tRNAs. **C** Volcano plot showing Differentially Expressed Genes (DEGs) in brain cortices between PHMG-p-exposed mice and control mice. **C** Volcano plot showing Differentially Expressed Genes (DEGs) in brain cortices between PHMG-p-exposed mice and control mice. The x-axis represents log₂ (fold change) and the y-axis represents log₁₀(q-value), where the q-value corresponds to the False Discovery Rate (FDR)-adjusted p-value. Genes meeting the significance criteria (|fold change| > 2 and q-value < 0.05) are highlighted in blue (downregulated) and red (upregulated). **D** Gene Ontology (GO) analysis of Biological Processes (BP) showing upregulated and downregulated processes. **E** Gene Ontology (GO) analysis of Cellular Components (CC) showing upregulated and downregulated components

We sought to establish comprehensively assess the gene expression profile to understand the molecular mechanisms underlying cortical modifications following PHMG-p administration. RNA extracted from cortical specimens exposed to PHMG-p underwent sequencing via QuantSeq 3’ mRNA-Seq technology and NextSeq 500/550 platforms. A total of 34 differentially expressed genes were identified (fold change > 2, q-value < 0.05), comprising 15 downregulated and 19 upregulated genes (Fig. 5A). Among these, genome-wide analysis revealed decreased expression of seven mitochondrial tRNAs—TrnP, TrnM, TrnL2, TrnV, TrnS2, TrnL1, and TrnT (Fig. 5B and C). As tRNAs play a central role in mRNA translation and maintenance of cellular proteostasis, their downregulation may reflect compromised translational capacity in cortical neurons following PHMG-p exposure.

Gene Ontology (GO) enrichment analysis was subsequently conducted to characterize the functional significance of the observed transcriptional changes. Downregulated genes were significantly enriched in biological processes associated with transcriptional regulation, CNS neuronal differentiation, and microtubule organization (Fig. 5D), all of which are essential for neuronal morphogenesis and synaptic maintenance. This transcriptomic profile aligns with the reduced dendritic spine density and decreased synaptic marker expression observed in PHMG-p-exposed mice (Fig. 4), supporting the notion that impairment of these transcriptional programs underlies the structural and synaptic deficits induced by PHMG-p. In contrast, upregulated genes were enriched in DNA double-strand break repair, cellular locomotion, and peroxisomal autophagy (Fig. 5D), indicative of the activation of stress and damage response mechanisms in the cortex. GO cellular component analysis corroborated these findings: downregulated transcripts were predominantly localized to dendritic compartments and transcriptional regulatory complexes, while upregulated transcripts were associated with cytoplasmic and endoplasmic reticulum-related components (Fig. 5E). Taken together, these results indicate that PHMG-p exposure suppresses transcriptional and dendritic gene programs critical for neuronal integrity while concurrently engaging stress-response pathways, providing a molecular basis for the cortical neuronal damage observed in this study.

## Discussion

**Fig. 6:**
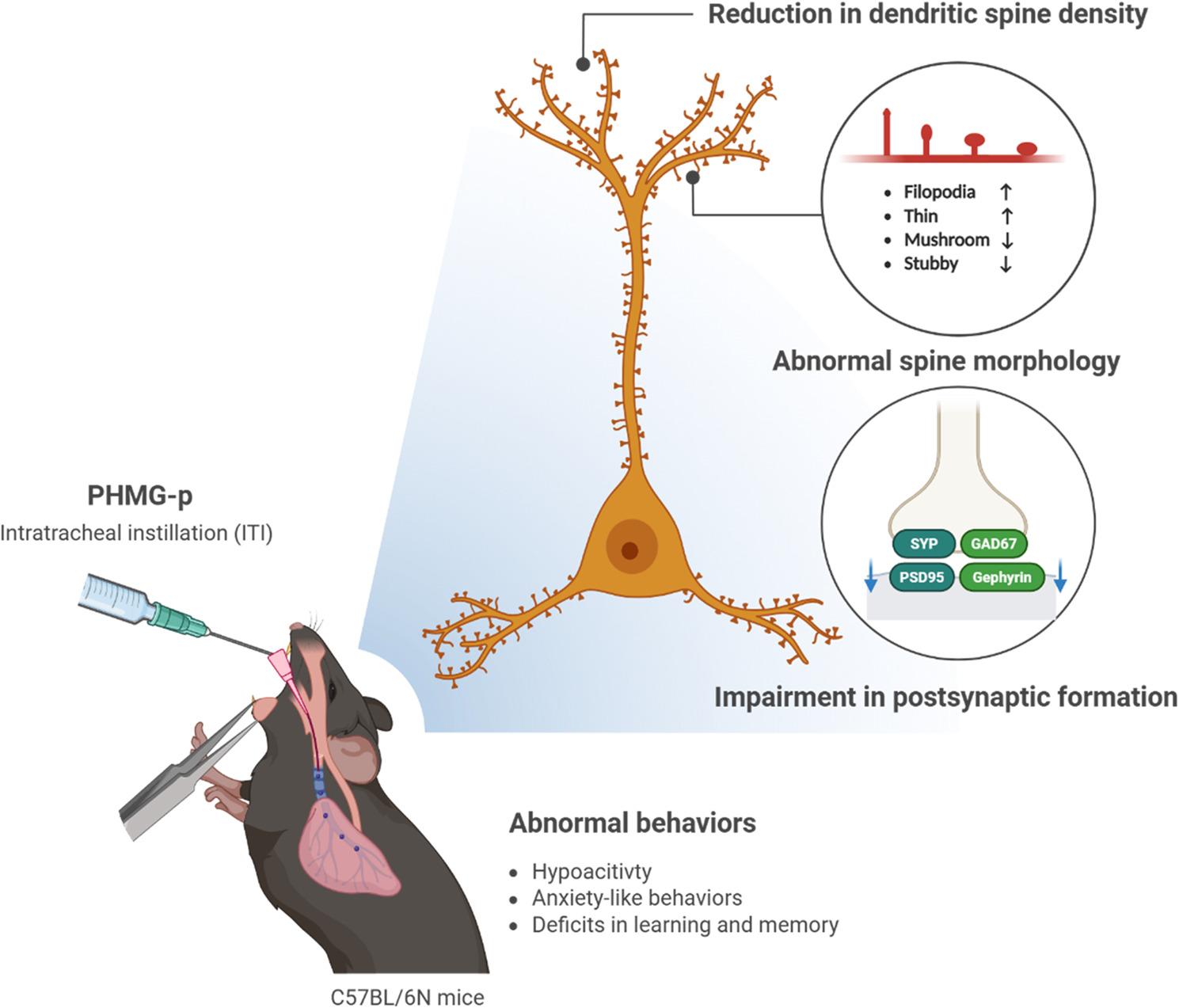
Summary. Schematic diagram illustrating the biological effects of prenatal PHMG-p exposure in mice

PHMG-p represents an antimicrobial sanitizing agent recognized for producing severe human toxicity responses. Nevertheless, its neurological impacts have not been well characterized. However, to date, the neurological effects of PHMG-p have been reported exclusively through epidemiological observations, and no experimental studies using in vitro or in vivo models have directly investigated the neurotoxic potential of PHMG-p. In the present investigation, we supplied evidence demonstrating that PHMG-p administration triggers behavioral abnormalities through the promotion of neural cell mortality and disruption of synaptic architecture within cortical brain regions (Fig. 6). Additionally, our findings offer new understanding regarding the structural and biochemical mechanisms underlying PHMG-p-induced neuronal death and synaptic dysfunction.

In this study, the experimental dose was selected to induce reproducible PHMG-p–mediated lung injury in mice, and its relevance to potential human exposure was evaluated based on reported indoor air concentrations during humidifier disinfectant use. Lee et al. (2017) reported an arithmetic mean indoor concentration of 8.26 ± 12.18 µg/m³ [28]. Using this value, the potential daily inhalation dose was estimated assuming an average adult inhalation rate of 0.83 m³/h, an exposure duration of 20 h/day, reflecting the fact that individuals typically spend the majority of their time indoors (U.S. EPA Exposure Factors Handbook) [29], a respiratory deposition fraction of 0.22 predicted by the MPPD model, and a body weight of 60 kg. Based on these parameters, the estimated human inhalation dose was approximately 0.05 µg/kg/day.

The dose used in the present study (0.9 mg/kg) is therefore substantially higher than the estimated environmental exposure level. However, this approach is consistent with conventional toxicological practice, as environmentally relevant exposure levels often produce minimal or undetectable biological responses within the limited duration of animal experiments. Higher doses are therefore commonly used to induce measurable pathological responses and to investigate the underlying mechanisms of toxicity. In the present study, the selected dose was intended to establish a reproducible PHMG-p–induced lung injury model and to evaluate potential downstream neurological effects. In addition, humidifier disinfectant users were likely exposed to PHMG-p repeatedly and for prolonged periods in indoor environments, which may further increase cumulative inhalation exposure.

A properly regulated innate immune response is crucial for combating immune challenges, while reducing the risk of an excessive, uncontrolled pro-inflammatory reaction [30]. The brain has long been recognized as the central regulator of body functions, including organ activity, nutrient preference, and metabolism [31–33]. The role of the brain in sickness-related responses, such as fever, elevated plasma corticosterone levels, increased pain sensitivity, and suppression of social and feeding behaviors, is well established [34]. PHMG-p administration results in substantial respiratory tissue damage, characterized by pulmonary inflammatory processes and tissue scarring [35]. Furthermore, PHMG-p induces cell cycle arrest at the G1/S checkpoint and promotes programmed cell death via the ROS/ATM/p53 signaling cascade in pulmonary epithelial tissue [36]. In our study, PHMG-p administration stimulated the infiltration of inflammatory cells including neutrophils, leading to macrophage activation within lung tissue. While the precise underlying mechanisms are not fully understood, respiratory pathologies characterized by specific inflammatory mediator patterns may influence neurological function via pulmonary-cerebral communication pathways, where oxygen deprivation and widespread inflammation serve as critical factors in compromising neural activity and physiological balance [37]. In the present investigation, PHMG-p administration caused neural cell mortality within cortical brain regions. Prior research has indicated that specific forms of neuronal death involve similar pathological processes as those observed in severe respiratory tissue injury [38]. PHMG-p exposure stimulates GFAP protein expression in cerebral cortical tissue, where increased GFAP levels indicate glial cell activation and reactive gliosis associated with neural degeneration [39]. These results indicate that PHMG-p promotes brain inflammation and neural cell death through mechanisms involving pulmonary tissue damage.

Neural connections facilitate immediate responses to motor, behavioral, and emotional signals [40, 41]. Activity-dependent synaptic adaptability represents a mechanism that alters the intensity of neural communication and serves essential functions in information storage and retention processes [42]. Therefore, impairments in synaptic operations can result in significant dysfunction of critical neurological mechanisms [43]. Disrupted synaptic integrity and function are established as cardinal pathological features underlying numerous progressive neurological diseases such as Alzheimer’s disease, amyotrophic lateral sclerosis, Parkinson’s disease, and Huntington’s disease [44]. Pulmonary endothelial amyloid deposits demonstrate neurotropic characteristics and decrease dendritic spine density in neurons [45]. In our investigation, PHMG-p administration compromised cognitive adaptability through reductions in neuronal populations, synaptic connections, and synaptic dimensions within cortical regions. Additionally, respiratory inflammation triggered by silica particle exposure in mice correlates with hippocampal inflammation, synaptic deterioration, and cognitive deficits [46]. We also observed that PHMG-p administration modified synaptic development in cortical tissue and resulted in behavioral abnormalities, including decreased activity, anxious responses, compromised spatial cognition and novel spatial exploration, and diminished motor function. The locomotion with open field test and rotarod test suggests that PHMG-p exposure impairs locomotor activity and motor coordination [47, 48]. In addition, Anxiety-like behavior can also be inferred from locomotor patterns in the open-field test, where increased time spent in the peripheral zone reflects heightened anxiety [49]. Consistent with this, mice exposed to PHMG-p showed increased time spent in the peripheral areas. Furthermore, anxiety-like behavior was also supported by the elevated plus maze results, where increased time spent in the closed arms is indicative of increased anxiety [50]. Taken together, these findings suggest that PHMG-p exposure induces anxiety-like behavioral alterations. Furthermore, PHMG-p–exposed mice showed reduced preference for the novel arm in the Y-maze task. This finding suggests reduced exploration and recognition of the novel environment rather than an effect of locomotor impairment. These behavioral alterations are consistent with previous study in Alzheimer’s disease (AD) models [51]. Importantly, the behavioral deficits observed in the present study can be directly linked to the specific synaptic and cellular alterations documented in our analyses. The reduced locomotor activity and anxiety-like behavior detected in the open field and elevated plus maze tests (Fig. 2) are consistent with the loss of cortical neurons and reactive astrogliosis demonstrated by decreased NeuN and increased GFAP expression (Fig. 3). Furthermore, the impaired spatial memory observed in the Y-maze and the motor coordination deficits detected in the rotarod test correspond with the reduced dendritic spine density and altered spine morphology (Fig. 4A–C), as well as the decreased excitatory synaptic marker expression (VGLUT) and reduced levels of pre- and post-synaptic proteins (SYP, PSD95) (Fig. 4D–L). These converging lines of evidence establish a coherent mechanistic framework in which PHMG-p-induced pulmonary inflammation leads to cortical neuronal damage, synaptic disruption, and ultimately behavioral dysfunction. These observations indicate that PHMG-p produces behavioral abnormalities through alterations in synaptic development.

Transfer RNA (tRNA) molecules serve fundamental functions in comprehensive protein synthesis processes [52]. Perturbations in transcriptional and translational regulatory networks that impair mRNA translation processes are established pathogenic mechanisms underlying neurological conditions including Fragile X syndrome, Rett syndrome, Parkinson’s disease, and Alzheimer’s disease [53–55]. In our current investigation, PHMG-p administration decreased tRNA concentrations in cortical brain tissue. The expression patterns, functional efficiency, and molecular stability of particular tRNAs correlate with neurodevelopmental and cognitive impairments [56]. Significantly, tRNA molecules have been identified in association with ribosomal complexes within dendritic spines [57]. Collectively, these results indicate that PHMG-p disrupts tRNA production, potentially resulting in behavioral abnormalities and modified synaptic architecture.

The present study has a limitation that should be acknowledged. Only a single dose of PHMG-p (0.9 mg/kg) was employed, precluding dose-response characterization and limiting the ability to establish threshold concentrations for neurotoxic effects. Future studies should address these limitations to further elucidate the neurotoxic mechanisms of PHMG-p. Dose-response studies employing multiple concentrations, including environmentally relevant low doses with chronic exposure paradigms, are needed to better approximate real-world human exposure conditions. Additionally, in vitro studies using human iPSC-derived neuronal models could complement the in vivo findings and enhance the translational relevance of the observed neurotoxic effects. Given that humidifier disinfectant exposure affected a large number of individuals in South Korea, including children and pregnant women, further investigation into the long-term neurological consequences of PHMG-p exposure and its potential impact on neurodevelopment is of significant public health importance.

In conclusion, as illustrated in the proposed mechanistic model (Fig. 6), intratracheal administration of PHMG-p in C57BL/6 N mice induces a reduction in dendritic spine density with altered spine morphology, accompanied by decreased expression of both excitatory and inhibitory synaptic proteins. These structural and molecular alterations in cortical neurons ultimately manifest as behavioral dysfunction, including hypoactivity, anxiety-like behaviors, and deficits in learning and memory. Our findings provide the first experimental evidence that PHMG-p induces neurotoxicity beyond its well-established pulmonary effects.

## Supplementary Information

Supplementary Material 1.

## Data Availability

The supporting materials can be obtained upon request via email to the corresponding author.

## Abbreviations

PHMG-p: Polyhexamethylene guanidine phosphate
tRNA: Transfer RNA
CNS: Central nervous system
BALF: Bronchoalveolar lavage fluid
PBS: Phosphate-buffered saline
NeuN: Neuronal nuclei (Fox-3, Rbfox3, or Hexaribonucleotide Binding Protein-3)
GFAP: Glial fibrillary acidic protein
VGLUT: Vesicular glutamate transporter
VGAT: Vesicular GABA transporter
Bax: BCL2 Associated X
Bcl2: B-cell lymphoma 2
SYP: Synaptophysin
PSD95: Postsynaptic density protein 95
GAD67: Gamma-aminobutyric acid (GABA) decarboxylase 67
AD: Alzheimer’s disease
ITI: Intratympanic injection
H&E: Hematoxylin and eosin
TrnP: tRNA-Proline
TrnM: tRNA-methionine
TrnL2: tRNA-leucine2
TrnV: tRNA-valine
TrnS2: tRNA-serine
TrnL1: tRNA-leucine1
TrnT: tRNA-threonine
GO: Gene Ontology
BP: Biological process
DNA: Deoxyribo nucleic acid
ROS: Reactive oxygen species
ATM: Ataxia-telangiectasia mutated kinase
p53: Tumor protein 53
