# Supplementary figures and images for: Polyhexamethylene guanidine phosphate exposure induces abnormal behaviors by disrupting synaptic formation and activity in the cerebral cortex
Source: J Neuroinflammation. 2026 Apr 23;23:190. doi: 10.1186/s12974-026-03832-0 (PMC13248417; doi:10.1186/s12974-026-03832-0)

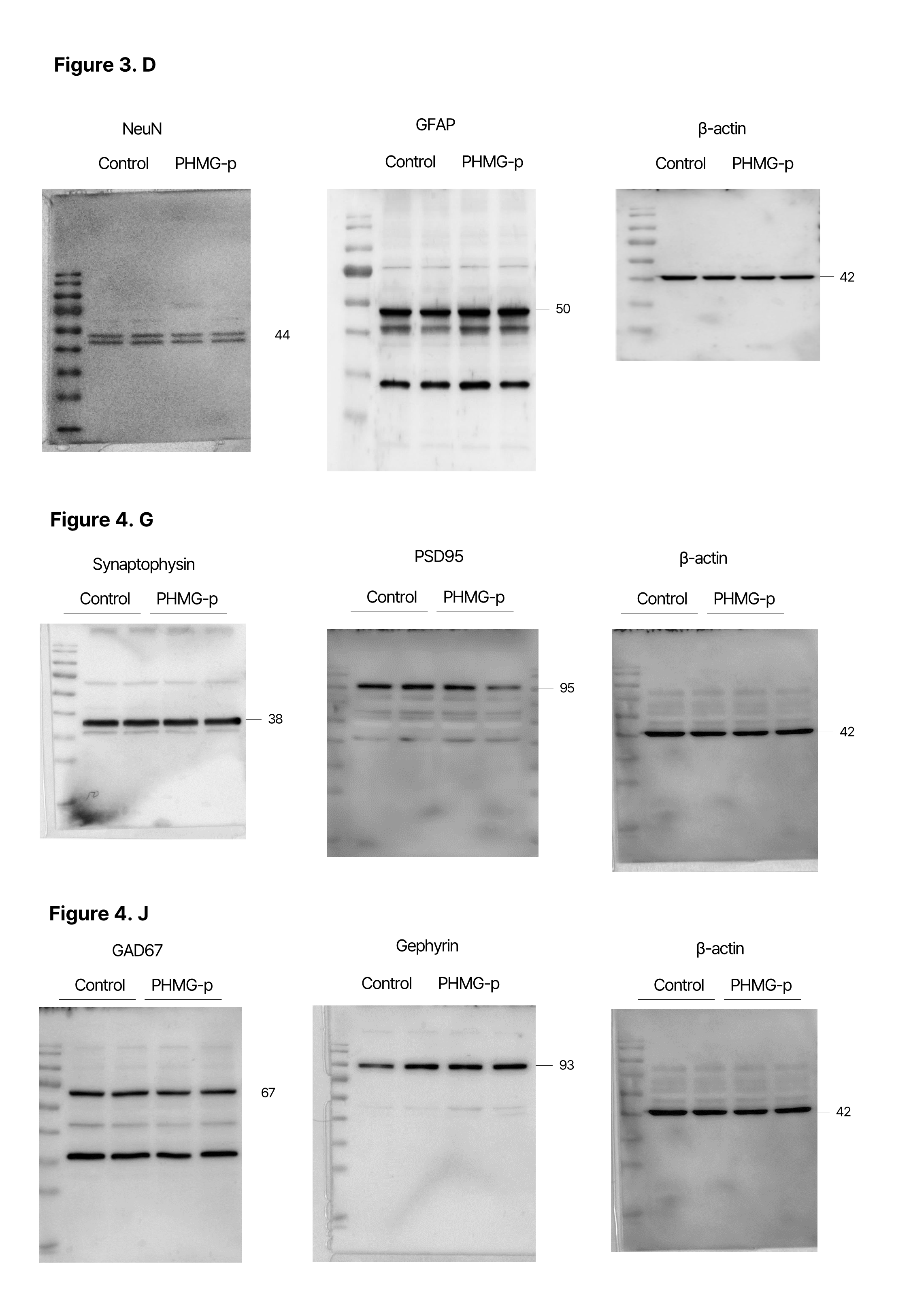

Supplement: Supplementary file 1 — Supplementary Material 1. [file 12974_2026_3832_MOESM1_ESM.tif]
